# Staged Endovascular Treatment With Stent-Assisted Coiling Followed by Flow Diversion for Ruptured Intracranial Dissecting Aneurysms: A Report of Two Cases

**DOI:** 10.7759/cureus.115746

**Published:** 2026-09-03

**Authors:** Yushi Nagano, Jun Sugata, Takuichiro Higashi, Hitoshi Yamahata, Ryosuke Hanaya

**Affiliations:** 1 Department of Neurosurgery, Kagoshima University Graduate School of Medical and Dental Sciences, Kagoshima, JPN

**Keywords:** flow diversion, ruptured intracranial dissecting aneurysm, staged endovascular treatment, stent-assisted coiling, subarachnoid hemorrhage

## Abstract

Ruptured intracranial dissecting aneurysms are challenging to treat when parent artery sacrifice is unsuitable or technically difficult. Although flow diverters (FDs) enable definitive arterial reconstruction, their use during acute subarachnoid hemorrhage is complicated by the need for dual antiplatelet therapy. We report two patients with ruptured dissecting aneurysms of the internal carotid artery and vertebral artery who underwent a staged endovascular strategy. Stent-assisted coiling (SAC) was performed in the acute phase to protect against rebleeding while preserving the parent artery. Both lesions subsequently demonstrated early recurrence: case 1 showed aneurysm enlargement with rebleeding and later recurrent filling, whereas case 2 developed a new focal dilatation at the proximal stent edge. FD placement was subsequently performed to reconstruct the diseased parent artery. Staged SAC, followed by FD placement, may provide a practical option for selected ruptured dissecting aneurysms by separating immediate protection against rebleeding from definitive parent artery reconstruction.

## Introduction

Ruptured intracranial dissecting aneurysms carry a high risk of acute-phase rebleeding, which is associated with catastrophic outcomes [1]. Parent artery occlusion (PAO) has traditionally been considered a standard treatment because exclusion of the diseased arterial segment can eliminate blood flow into the fragile dissected lesion and thereby prevent rebleeding [2]. However, PAO may be unsuitable or technically difficult when adequate collateral circulation is lacking, critical perforating branches are involved, or the lesion arises from the internal carotid artery [3].

In such situations, reconstructive treatment with preservation of the parent artery may be required. Stent-assisted coiling (SAC) provides a scaffold for coil embolization while maintaining parent artery patency; however, it does not necessarily reconstruct the entire diseased arterial segment, and residual entry flow or progression of the dissection may result in aneurysm regrowth or recurrence [4,5]. In contrast, a flow diverter (FD) provides higher metal coverage across the diseased segment, reducing blood flow into the aneurysm and promoting endothelialization along the parent artery, thereby facilitating vascular reconstruction. Nevertheless, acute-phase FD deployment after subarachnoid hemorrhage (SAH) is constrained by the need for dual antiplatelet therapy (DAPT) and the associated risk of hemorrhagic complications [6-9].

A staged strategy using SAC for acute protection, followed by FD placement for definitive reconstruction, has been reported; however, the clinical role of this strategy and the patterns of early recurrence after SAC remain incompletely characterized. In the present report, two patients demonstrated distinct patterns of early recurrence after acute-phase SAC: aneurysm enlargement with rebleeding in one patient and a new focal dilatation at the proximal stent edge in the other. We describe how subsequent FD placement achieved definitive parent artery reconstruction and discuss the rationale for this selective staged strategy.

## Case presentation

Case 1

A 47-year-old female was admitted after sudden-onset impaired consciousness. On arrival, she had a Japan Coma Scale (JCS) score of 30 [10] and a Glasgow Coma Scale (GCS) score of 11 (E3V3M5) [11], corresponding to the World Federation of Neurosurgical Societies (WFNS) grade II [12]. Non-contrast head CT showed diffuse SAH in the basal cisterns (Fisher group 3) (Figure 1A) [13]. Although initial CT angiography did not identify the bleeding source, digital subtraction angiography (DSA) performed the following day revealed a new small aneurysm in the C2 segment of the right internal carotid artery, consistent with a ruptured dissecting aneurysm (Figure 1B).

To avoid ischemic complications from parent artery occlusion, off-label acute-phase SAC was planned. After placement of the arterial sheath, aspirin 200 mg and clopidogrel 300 mg were administered as loading doses through a nasogastric tube. Intravenous heparin (2,000 units) was then administered, followed by an adjusted continuous heparin infusion with serial activated clotting time (ACT) measurements to maintain an ACT of >200 seconds. A low-profile visualized intraluminal support (LVIS) stent (4.0 × 22 mm; Terumo, Tokyo, Japan) was deployed using the semi-jailing technique, with coil embolization covering the dissected segment (Figures 1C, 1D). No immediate perioperative complications occurred.

**Figure 1:**
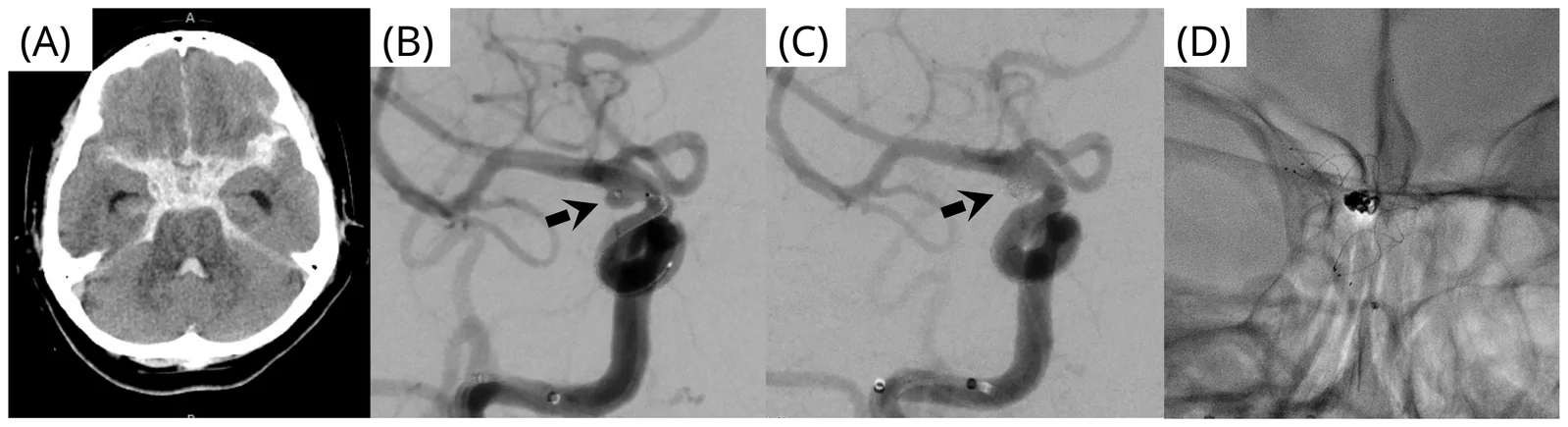
Initial imaging and stent-assisted coiling in case 1. (A) Initial non-contrast computed tomography showing diffuse subarachnoid hemorrhage in the basal cisterns. (B) Initial angiography showing a ruptured blister-like aneurysm at the C2 segment of the right internal carotid artery (arrow). (C) Angiography after stent-assisted coiling showing aneurysm occlusion with preservation of the parent artery. (D) Unsubtracted image showing the deployed stent and coils.

Follow-up angiography showed mild intra-aneurysmal recanalization. On day 26, the patient developed sudden neurological deterioration, and head CT confirmed rebleeding. Emergency DSA revealed significant aneurysm enlargement (Figure 2A); urgent retreatment was therefore performed with additional coil embolization using the trans-cell technique (Figure 2B). The patient’s neurological condition gradually improved.

Follow-up angiography on day 59 revealed minor recurrent contrast filling within the aneurysm (Figure 2C). To achieve durable vascular reconstruction, FD placement was performed on day 72. Platelet reactivity was assessed using the VerifyNow P2Y12 assay before FD placement, with a P2Y12 reaction unit (PRU) value of 134. During the FD procedure, 3,000 units of intravenous heparin were administered after placement of the arterial sheath, followed by an adjusted continuous heparin infusion with serial ACT measurements to maintain an ACT of >250 seconds. Two pipeline embolization devices (3.25 × 16 mm and 3.5 × 18 mm; Medtronic, Minneapolis, MN) were deployed in a telescoping fashion to fully cover the previously treated segment, extending beyond both the distal and proximal edges of the LVIS stent (Figures 2D, 2E). DAPT with aspirin 100 mg/day and clopidogrel 75 mg/day was continued for six months after FD placement. After angiographic confirmation of a favorable course at six months, aspirin was discontinued, and clopidogrel monotherapy was continued. Clopidogrel was subsequently tapered and discontinued after the one-year follow-up confirmed no recurrence.

Follow-up DSA two months after FD placement demonstrated complete occlusion (O’Kelly-Marotta (OKM) grade D) (Figure 2F) [14]. Follow-up DSA at 12 months confirmed persistent complete occlusion without recurrence, and the patient had a modified Rankin Scale (mRS) score of 0 [15]. The timeline of case 1 is presented in Table 1.

**Figure 2:**
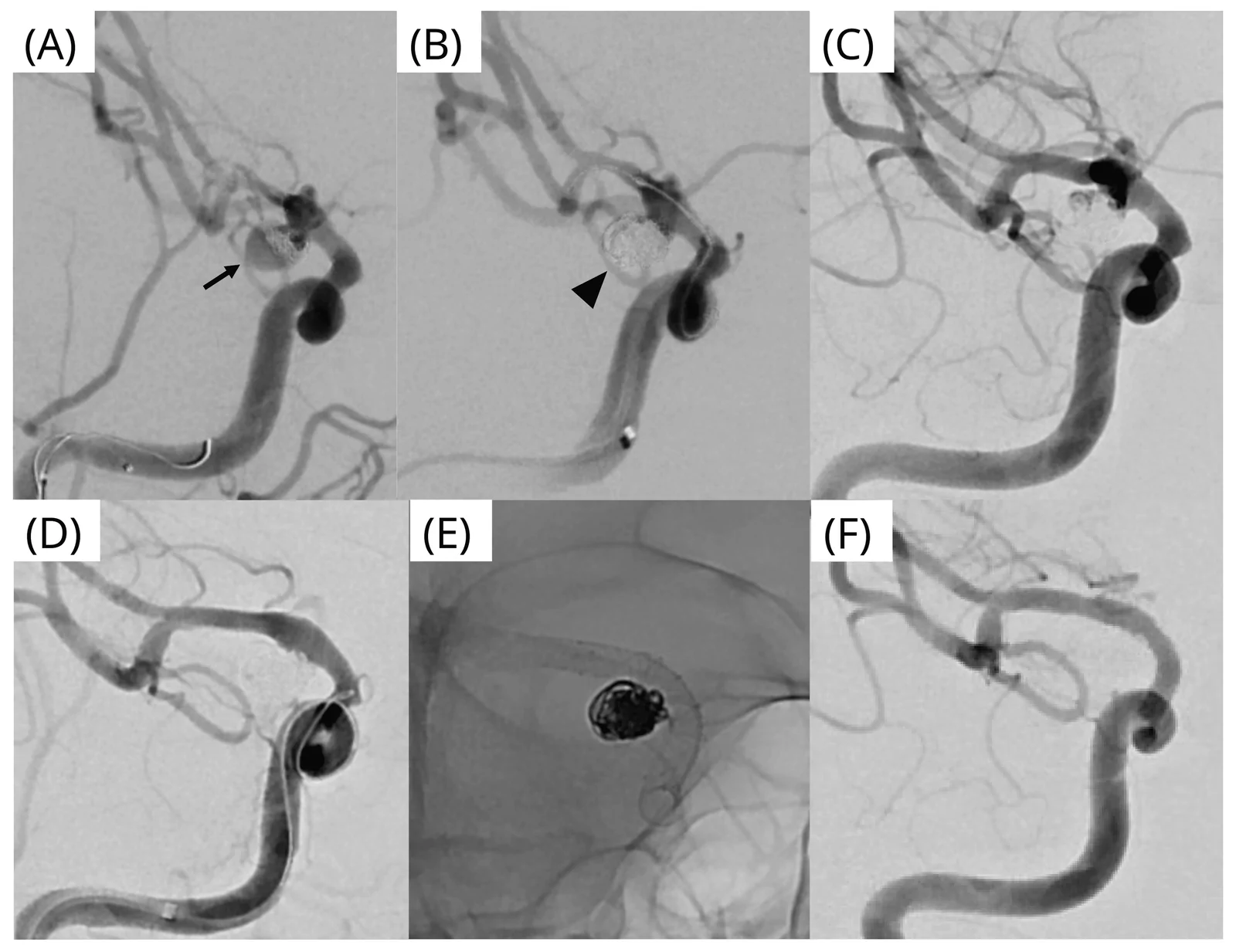
Recurrence and subsequent flow diverter treatment in case 1. (A) Follow-up angiography showing recurrent aneurysm filling after rebleeding (arrow). (B) Angiography after additional coil embolization showing temporary aneurysm occlusion. (C) Follow-up angiography showing recurrent aneurysm filling (arrowhead). (D) Angiography after flow diverter placement showing parent artery reconstruction. (E) Unsubtracted image showing the deployed flow diverter across the aneurysm segment. (F) Follow-up angiography at two months showing complete aneurysm occlusion with parent artery reconstruction.

**Table 1:** Timeline of case 1. Abbreviations: SAH, subarachnoid hemorrhage; DSA, digital subtraction angiography; SAC, stent-assisted coiling; FD, flow diverter; OKM, O’Kelly-Marotta; mRS, modified Rankin Scale.

Time	Clinical course
Day 0	SAH
Day 1	DSA and SAC
Day 26	Rebleeding and additional coil embolization
Day 59	Angiographic recurrence
Day 72	FD placement
2 months after FD	Complete occlusion (OKM grade D)
12 months	DSA: no recurrence; mRS 0

Case 2

A 57-year-old female presented with a sudden occipital headache. On arrival, her GCS score was 15 [11], corresponding to WFNS grade I [12]. Head CT showed SAH predominantly in the posterior fossa (Figure 3A). DSA revealed subtle wall irregularity of the right vertebral artery (VA), consistent with ruptured VA dissection (Figure 3B).

Because the contralateral VA was absent, PAO was precluded, and parent artery preservation was prioritized. The same acute-phase antiplatelet and anticoagulation protocol as in case 1 was used. After placement of the arterial sheath, aspirin 200 mg and clopidogrel 300 mg were administered as loading doses through a nasogastric tube. Intravenous heparin (2,000 units) was then administered, followed by an adjusted continuous heparin infusion with serial ACT measurements to maintain an ACT of >200 seconds. SAC was performed using an LVIS stent (4.5 × 23 mm; Terumo, Tokyo, Japan), with limited coiling via the semi-jailing technique (Figure 3C).

Although DSA on postoperative days 1 and 7 showed stable findings, follow-up DSA on day 21 revealed a new focal dilatation at the proximal edge of the stent (Figure 3D). Platelet reactivity was assessed using the VerifyNow P2Y12 assay before FD placement (PRU, 141). A pipeline embolization device (4.0 × 35 mm; Medtronic, Minneapolis, MN) was subsequently deployed on day 25 to fully cover the lesion (Figure 3E). During FD placement, 3,000 units of intravenous heparin were administered after placement of the arterial sheath, followed by an adjusted continuous heparin infusion with serial ACT measurements to maintain an ACT of >250 seconds.

Follow-up DSA two months after FD placement showed complete occlusion (OKM grade D) (Figure 3F) [14]. DAPT with aspirin 100 mg/day and clopidogrel 75 mg/day was continued for six months after FD placement. After favorable angiographic findings were confirmed at six months, aspirin was discontinued, and clopidogrel monotherapy was continued. Clopidogrel was subsequently tapered and discontinued after the 12-month follow-up confirmed no recurrence. The patient remained neurologically independent with an mRS score of 0 [15]. The timeline of case 2 is presented in Table 2.

**Figure 3:**
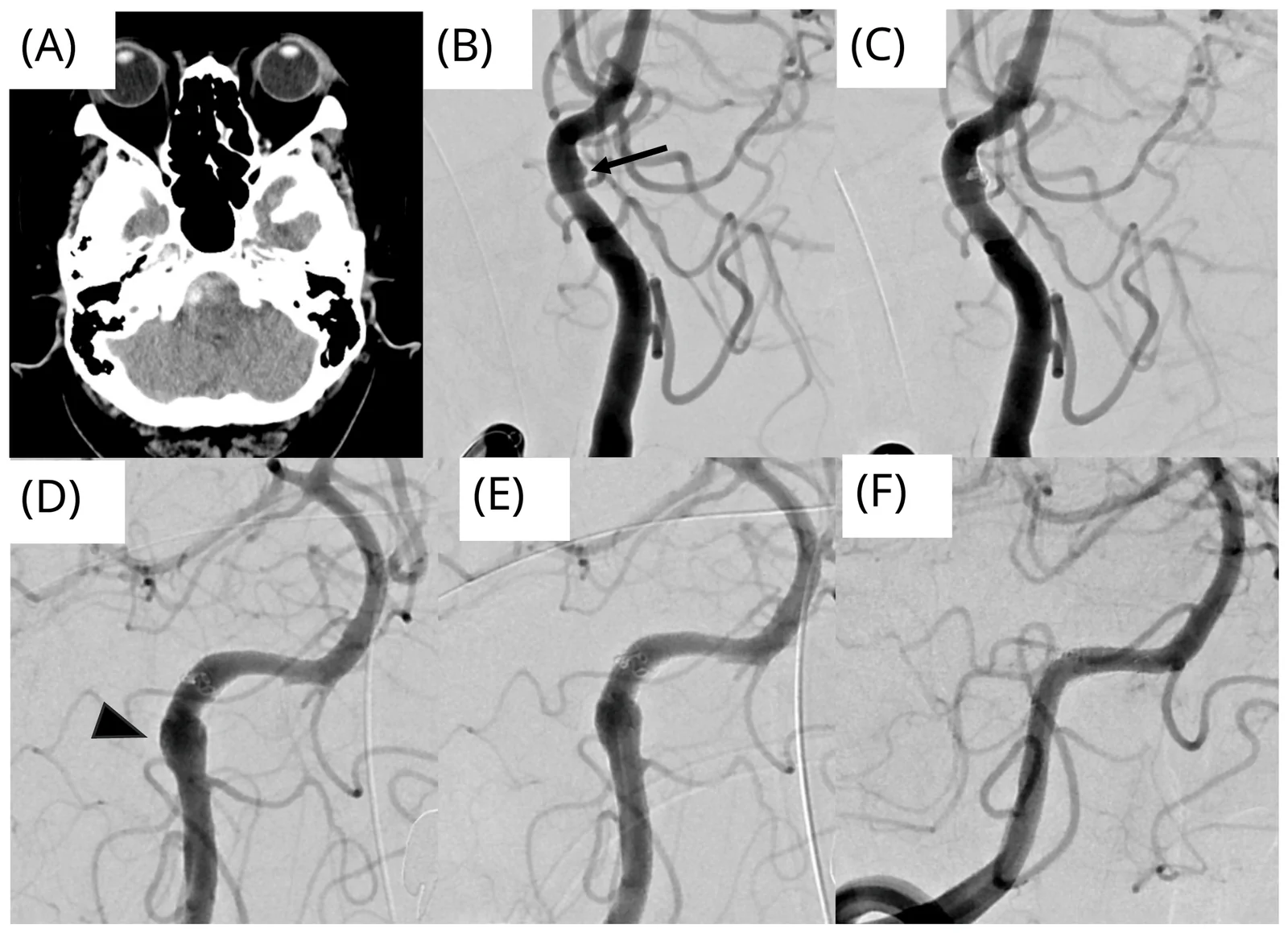
Initial imaging and staged endovascular treatment in case 2. (A) Initial non-contrast computed tomography showing subarachnoid hemorrhage predominantly in the posterior fossa. (B) Initial vertebral angiography showing subtle wall irregularity of the right vertebral artery, consistent with a dissecting lesion (arrow). (C) Angiography after stent-assisted coiling showing initial aneurysm occlusion with preservation of the parent artery. (D) Follow-up angiography on day 21 showing a new focal dilatation at the proximal edge of the previously deployed stent (arrowhead). (E) Angiography after flow diverter placement showing reconstruction of the parent artery. (F) Follow-up angiography two months after flow diverter placement showing complete aneurysm occlusion (O’Kelly-Marotta grade D).

**Table 2:** Timeline of case 2. Abbreviations: SAH, subarachnoid hemorrhage; SAC, stent-assisted coiling; FD, flow diverter; OKM, O’Kelly-Marotta; mRS, modified Rankin Scale.

Time	Clinical course
Day 0	SAH and SAC
Day 1 and day 7	Stable angiographic findings
Day 21	Proximal stent-edge recurrence
Day 25	FD placement
2 months after FD	Complete occlusion (OKM grade D)
12 months	mRS 0; no recurrence

## Discussion

The principal advantage of this staged strategy is that it separates immediate protection against rebleeding from durable vascular reconstruction. During acute-phase SAH, the dissected arterial wall is highly unstable and susceptible to catastrophic rebleeding [1]. Although SAC provides a scaffold for coil embolization and can secure the ruptured site, durable occlusion may remain difficult [4,5]. In case 1, the aneurysm enlarged and rebled after initial SAC, whereas in case 2, a new focal dilatation developed at the proximal stent edge within three weeks. These distinct patterns illustrate the dynamic nature of dissecting lesions and the limitations of SAC as definitive reconstruction. These cases also highlight the diagnostic challenge of ruptured dissecting aneurysms, as the causative lesion may be occult or subtle on initial vascular imaging. When no definite bleeding source is identified, but a dissecting aneurysm remains clinically suspected, careful repeat angiographic evaluation may be important for detecting dynamic morphological changes.

Previous reports have described FD placement for recurrence after SAC in ruptured dissecting aneurysms [16,17]. Our cases support this approach while emphasizing two potential mechanisms of early failure: regrowth of the aneurysm itself and recurrence at the edge of the previously deployed stent. Although the precise mechanisms cannot be determined from these two cases, the latter may reflect persistent entry flow or incomplete coverage of the diseased arterial segment [16]. FD treatment can address these limitations by providing higher metal coverage, reducing inflow, and promoting endothelialization and reconstruction of the parent artery.

The timing of the second stage is an important consideration. In our cases, conversion to FD treatment was considered when follow-up angiography demonstrated recurrent filling, aneurysm enlargement, or a new focal dilatation suggesting progression of the dissected segment despite initial SAC. The timing of FD placement was individualized according to the urgency of the angiographic findings, clinical stability, and the feasibility of maintaining adequate antiplatelet therapy. In case 2, FD placement was performed on day 25, after the acute phase had begun to resolve. A pre-existing braided stent such as the LVIS may also provide technical support for subsequent FD delivery and facilitate accurate deployment across the dissected segment. However, the optimal timing of conversion to FD treatment remains uncertain and should be individualized according to aneurysm behavior, the patient’s clinical condition, and the anticipated need for additional procedures.

Acute-phase FD placement has been reported for ruptured intracranial aneurysms [18,19], but hemorrhagic complications associated with DAPT remain an important concern [6,7]. SAC also requires adequate antiplatelet therapy; nevertheless, staged treatment may provide greater flexibility in perioperative antiplatelet management before definitive FD reconstruction. This consideration may be particularly relevant when external ventricular drainage, additional surgery, or other invasive procedures may be required during the acute SAH period.

Our proposed treatment concept is therefore selective rather than routine, as summarized in Figure 4. PAO should remain a primary consideration when it can be performed safely. When parent artery sacrifice is unsuitable or technically difficult, acute-phase SAC can be used as bridge therapy for immediate protection. Close angiographic surveillance is essential because early recurrence may occur. Stable lesions may be observed, whereas recurrent or dynamically enlarging lesions may be considered for FD placement in the subacute or delayed phase to achieve durable parent artery reconstruction.

**Figure 4:**
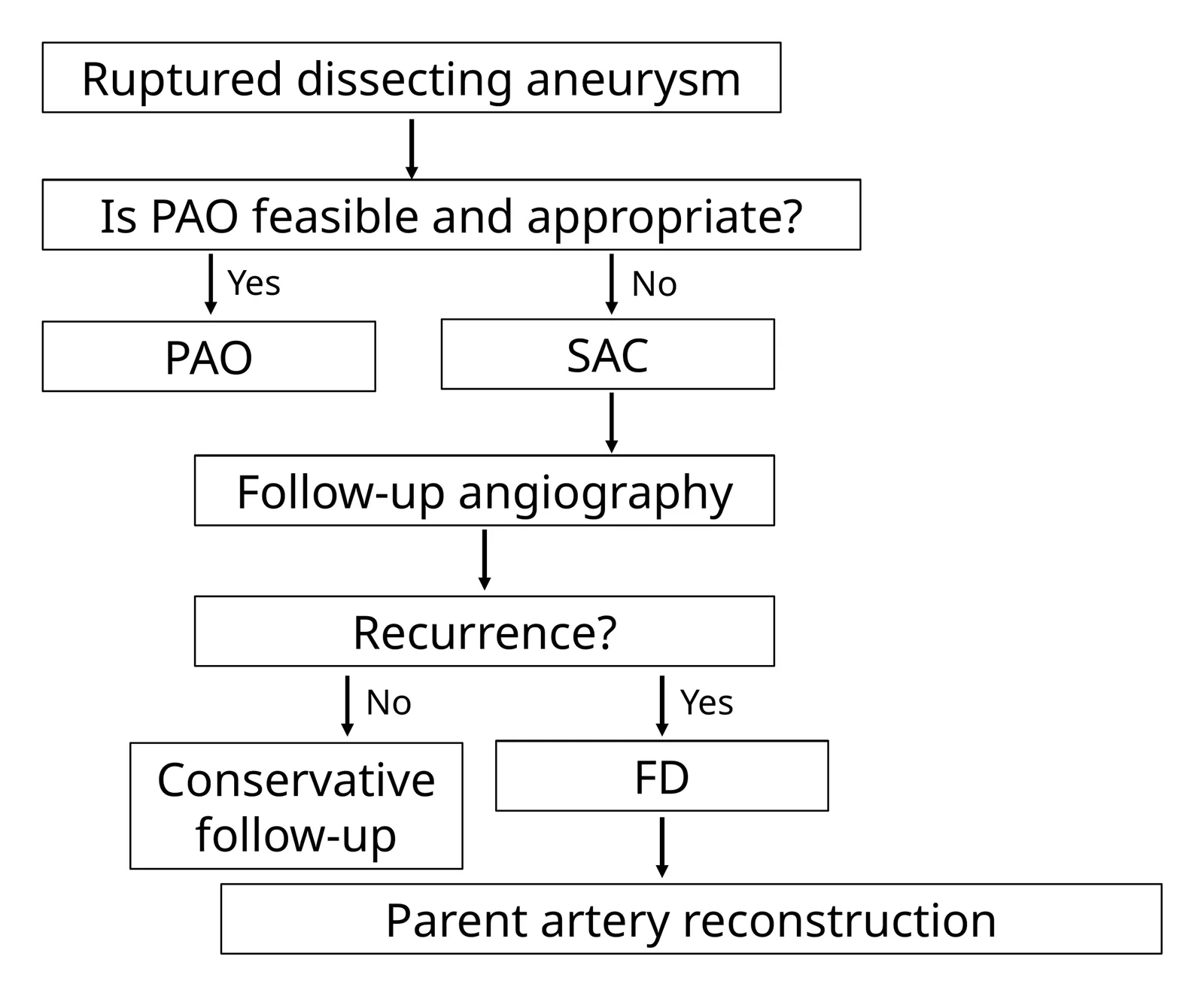
Proposed treatment concept for ruptured intracranial dissecting aneurysms. When parent artery occlusion (PAO) is unsuitable, technically difficult, or contraindicated, acute-phase stent-assisted coiling (SAC) may be used for initial protection against rebleeding. Follow-up angiography guides subsequent management: conservative follow-up for stable lesions or flow diverter (FD) placement for recurrent lesions to achieve durable parent artery reconstruction.

This report is limited by the small number of patients and the relatively short angiographic follow-up after FD placement. The proposed strategy and timing cannot therefore be generalized, and additional cases with longer follow-up are required to clarify patient selection and the optimal timing of FD conversion.

## Conclusions

Staged endovascular treatment with acute-phase SAC, followed by FD placement after recurrence, achieved initial protection against rebleeding and subsequent parent artery reconstruction in two patients with ruptured intracranial dissecting aneurysms. This strategy may be a practical option for carefully selected patients when parent artery sacrifice is unsuitable or technically difficult, provided that close angiographic follow-up is performed. Further studies with larger numbers of patients and longer follow-up are needed to determine the long-term durability and optimal role of this staged treatment strategy.
